# Can urinary 8-OHdG be a good indicator of vasospasm occurrence following subarachnoid hemorrhage?

**DOI:** 10.1186/cc10920

**Published:** 2012-03-20

**Authors:** K Ikeda, T Ikeda, H Taniuchi, S Suda, Y Ikeda, H Jimbo

**Affiliations:** 1 Tokyo Medical University, Hachioji Medical Center, Tokyo, Japan

## Introduction

There is substantial evidence to suggest that oxidative stress is associated with cerebral vasospasm following subarachnoid hemorrhage (SAH). Urinary 8-OHdG is the most common biomarker of DNA damage by oxidative stress. The aim of this study was to determine whether 8-OHdG is a good indicator of vasospasm occurrence following SAH.

## Methods

The subjects were 23 patients who received surgical clipping or endovascular coiling within 24 hours after the onset of SAH. We classified the patients according to the occurrence of angiographic vasospasm. We examined the urinary 8-OHdG levels with high-performance liquid chromatography for 10 days following SAH. The urinary 8-OHdG levels were adjusted according to serum creatinine levels.

## Results

The urinary 8-OHdG levels were elevated on day 2 compared with those on day 1 only in the vasospasm (+) group. The urinary 8-OHdG levels in the vasospasm (+) group were significantly higher than those in the nonvasospasm (-) group on days 1, 2, 8 and 9. Furthermore, we examined the correlations between the urinary 8-OHdG levels on admission to the ICU and the grades of the World Federation of Neurologic Surgeons and Fisher, but none were observed. *Discussion *An elevated urinary 8-OHdG level on day 2 was observed only in the vasospasm group. Therefore, we speculated that free radicals may have a role in inducing vasospasm in the early phase following SAH. The urinary 8-OHdG levels were higher in the vasospasm group than in the nonvasospasm group, but we did not find any correlation with severity of SAH. We suspect that the higher urinary 8-OHdG levels on days 8 and 9 in the vasospasm group indicated ischemic brain injury after vasospasm.

## Conclusion

We believe that oxidative stress has a role in the development of cerebral vasospasm and that urinary 8-OHdG may be a good indicator of vasospasm occurrence following SAH.

